# Comparative analysis and forecast of spinal cord injury burden in BRICS countries (1990–2021): insights from the Global Burden of Disease Study 2021

**DOI:** 10.3389/fpubh.2025.1642367

**Published:** 2025-10-16

**Authors:** Kongjun Yuan, Mengye Ou, Shaohui Chen, Tian Bai, Hailong Lin, Zhuqiang Luo, Yanli Gao, Xiaoqiang Chen, Sixiong Shi, Dan Zhu

**Affiliations:** ^1^Medical Affairs, The Sixth Affiliated Hospital of Jinan University (Dongguan Eastern Central Hospital), Dongguan, China; ^2^Dongguan Institute of Spine and Spinal Cord Injury, Dongguan, China; ^3^Personnel, The Sixth Affiliated Hospital of Jinan University (Dongguan Eastern Central Hospital), Dongguan, China

**Keywords:** spinal cord injury, burden of disease, BRICS countries, ARIMA model, etiology analysis

## Abstract

**Background:**

As major emerging economies, the BRICS (Brazil, Russia, India, China, and South Africa) face a significant and distinct burden of spinal cord injury (SCI). We systematically assessed trends in the burden of SCI and its leading causes from 1990 to 2021 and forecasted trends to 2031, providing evidence to optimize prevention and control strategies.

**Methods:**

Based on the Global Burden of Disease Study 2021 (GBD 2021), we calculated the age-standardized incidence and prevalence rate (ASIR, ASPR) of SCI in the BRICS, along with the estimated annual percentage change (EAPC) and average annual percentage change (AAPC). Furthermore, we analyzed the etiological composition of the SCI burden and developed an autoregressive integrated moving average (ARIMA) model to forecast its trends from 2022 to 2031.

**Results:**

From 1990 to 2021, the absolute number of SCI cases rose in all BRICS countries except Russia; however, age-standardized rates (ASRs) fell consistently (EAPC range: −2.15 to −0.13). South Africa demonstrated the most substantial reductions (AAPC: ASIR –1.59, ASPR −1.93), while China showed the modest declines (−0.23 and −0.09, respectively). The burden of SCI varied substantially by age and sex. Males bore a consistently higher burden, with peak risks shifting from younger to older females. The peak number of cases occurred earlier (20–35 years) in Brazil, Russia, and South Africa, but was notably delayed in China and India (50–54 years). The leading causes of SCI across the BRICS included falls (70% of cases in India), road injuries, and self-harm and interpersonal violence (35% of cases in South Africa). Based on ARIMA modeling, a continued decline in ASRs is projected for all member countries over the coming decade.

**Conclusion:**

The burden of SCI in the BRICS countries is influenced by demographic, socioeconomic, and policy-related factors. Although ASRs have shown improvement, the absolute number of cases continues to grow. This trend necessitates tailored preventive strategies that address specific age, sex, and etiological factors, supported by enhanced international health cooperation, to mitigate the global burden of SCI.

## Introduction

Spinal cord injury (SCI) is one of the most severe outcomes of spinal injury, leading to permanent and often devastating neurological impairments in motor, sensory, and autonomic functions (1–4). The etiology of SCI is broadly classified into traumatic (e.g., traffic accidents, falls, violence) or non-traumatic categories (e.g., tumors, degenerative diseases) (5). SCI poses a substantial global disease burden. The GBD 2019 study reported that over 20 million individuals lived with SCI in 2019, with more than 900,000 new cases, contributing to 6.2 million years lived with disability (YLD) (6). Owing to the largely irreversible nature of neuronal injury, current therapeutic strategies cannot achieve complete neurological recovery. Therefore, clinical management primarily focuses on surgical interventions, including decompression and stabilization (7–9), and non-surgical approaches, notably early steroid administration to mitigate edema (7, 10). However, these treatments remain palliative, aimed at preventing secondary deterioration rather than achieving neural repair. This reality leaves many patients with permanent disabilities that severely impact their physical and psychological health and impose a heavy socioeconomic burden. Given the limitations of these therapeutic options, comprehensive multidisciplinary rehabilitation encompassing, including physical, occupational, and psychological therapy, in addition to assistive technology, is crucial for improving functional outcomes and quality of life. Thus, primary prevention remains the most critical strategy in addressing SCI.

The epidemiological profile of SCI varies considerably worldwide. Whereas high-income regions like North America and Australia report ASIRs of 22 and 14 per 100,000 respectively, rates in developing countries range from 0.2 to 13.0 per 100,000. This disparity stems from divergent socioeconomic conditions, infrastructure, surveillance systems, and etiological factors (6, 11, 12). Such heterogeneity is especially pronounced and of strategic importance among the BRICS (Brazil, Russia, India, China, and South Africa). As emerging economies that collectively account for 42% of the world’s population, the BRICS countries share rapid development. They also face common challenges, such as industrial expansion, environmental pollution, and shifting demographic patterns. This unique position means that these factors may directly or indirectly influence the risk and burden of SCI (13). For instance, air pollutants like PM₂.₅ and NO₂ can indirectly increase the risk of traffic accidents by reducing visibility. Furthermore, rapid urbanization may heighten population exposure to traffic-related incidents, while industrial expansion can lead to more occupational injuries. However, despite these shared challenges, profound differences in economic structures, healthcare systems, and policies among these countries likely result in distinct national patterns of SCI. A comparative analysis is therefore essential to clarify these drivers and guide public health interventions. Using GBD 2021 data, we pursued three specific aims: (1) analyze temporal trends in age-standardized incidence and prevalence rates (ASIR and ASPR) of SCI across BRICS (1990–2021); (2) compare causative factors to pinpoint national priorities; and (3) forecast the burden to 2031 using an autoregressive integrated moving average (ARIMA) model. Ultimately, by elucidating the dynamic interplay between socioeconomic development and SCI epidemiology, this study seeks to provide an evidence-based framework for optimizing prevention and policy in BRICS and other similar rapidly transitioning economies.

## Data and methods

Data on the incidence, prevalence, and other burden of SCI in BRICS countries were derived from the GBD 2021. Coordinated by the Institute for Health Metrics and Evaluation (IHME), the GBD 2021 systematically quantifies health loss from 371 diseases and injuries and 88 risk factors across 204 countries and territories. Metrics include incidence, prevalence, and disability-adjusted life years (DALYs), among others. All related data are publicly available through the Global Health Data Exchange (GHDx) platform (14, 15). Within the GBD framework, injuries are classified both by cause (e.g., falls, road traffic injuries) and by nature (e.g., spinal cord injury) (16). As one causative injury can lead to multiple natures of injury, and vice versa, the GBD study correlates them using a severity rating system to estimate the burden of disease. Case definitions for SCI are rigorously based on International Classification of Diseases (ICD-9 and ICD-10) codes (17).

To quantify the temporal trend in the age-standardized rate (ASR) of SCI from 1990 to 2021, we calculated the estimated annual percentage change (EAPC). For a comprehensive assessment, we initially estimated the EAPC under the assumption of a consistent linear trend over the entire period. Subsequently, we applied Joinpoint regression (JPR) analysis to detect potential inflection points and calculated the average annual percentage change (AAPC) to characterize the overarching trend across identified temporal segments. This was done by fitting a linear regression model to the natural logarithm of the ASR against calendar year. The model is defined as: ln(ASR)=α+β×year+ε, where *α* is the intercept, *β* is the slope coefficient indicating the direction and magnitude of the trend, year is the calendar year, and *ε* is the error term. The EAPC and its 95% confidence interval (CI) were then derived from the slope *β* using the formula: $\text{EAPC}=100\times e^{\beta }-1$ (18). Trends were interpreted based on the EAPC and its 95% CI as follows: an upward trend was defined as an EAPC with a lower 95% confidence limit >0; a downward trend was defined as an EAPC with an upper 95% confidence limit <0; and the trend was considered statistically non-significant if the 95% CI contained zero.

JPR is a commonly used model for statistical analysis of time-series data (19, 20). This algorithm fits a series of connected linear segments to the data by determining the optimal number and locations of points where the trend changes significantly (i.e., joinpoints). Within each segment, a linear regression model was fitted to the natural logarithm of the ASR (ϒ) against the calendar year (χ), formulated as: ln(y)=α+βx+ε, in which y denotes the ASR and χ denotes the calendar year. The overall summary trend from 1990 to 2021 was quantified by the average annual percentage change (AAPC), which is derived as the geometrically weighted average of the slope coefficients (*β*) from all segments. The statistical significance of the AAPC was assessed by its 95% CI: an upward trend was concluded if the AAPC and the lower bound of its 95% CI were >0; a downward trend was concluded if the AAPC and the upper bound of its 95% CI were <0; and a non-significant trend was indicated if the 95% CI contained zero.

Forecasting of the ASR of SCI from 2022 to 2031 was performed using an ARIMA model. The ARIMA framework, denoted by the parameters (p, d, q), integrates autoregressive (AR), differencing (I), and moving average (MA) components to model and predict time series data. The analysis was conducted in R software (version 4.4.2) using the “forecast” package. The optimal combination of (p, d, q) parameters was automatically selected using the “auto.arima” function (21, 22), which minimizes the Akaike Information Criterion (AIC) and Bayesian Information Criterion (BIC), with lower values indicating a superior model fit. The adequacy of the fitted model was verified using the Ljung–Box test (at lags 6 and 12) to assess whether the residual series exhibited white noise properties (i.e., were uncorrelated). A *p*-value > 0.05 indicates failure to reject the null hypothesis of independence, supporting the adequacy of the model specification. Data from 1990 to 2018 were used as the training set to build the model, while data from 2019 to 2021 served as the test set for external validation. Model performance was evaluated using a test set comprising data from 2019 to 2021. Predictive accuracy was quantified by calculating the root mean square error (RMSE), mean absolute error (MAE), and mean absolute percentage error (MAPE), with lower values for each metric indicating higher forecast precision.

Epidemiological data were collated and organized using Microsoft Excel 2019. The raw data were then age-standardized using the GBD 2021 Global Standard Population to calculate the ASR. Subsequently, temporal trends in ASR were analyzed by estimating the AAPC and identifying potential inflection points using JPR Software (version 5.2.0.0). Following the trend analysis, forecasting was performed by applying an ARIMA model in R (version 4.4.2). All data visualizations were generated using Origin (version 2024).

## Results

### Incidence and prevalence of SCI in BRICS countries, 1990–2021

**Figure 1:**
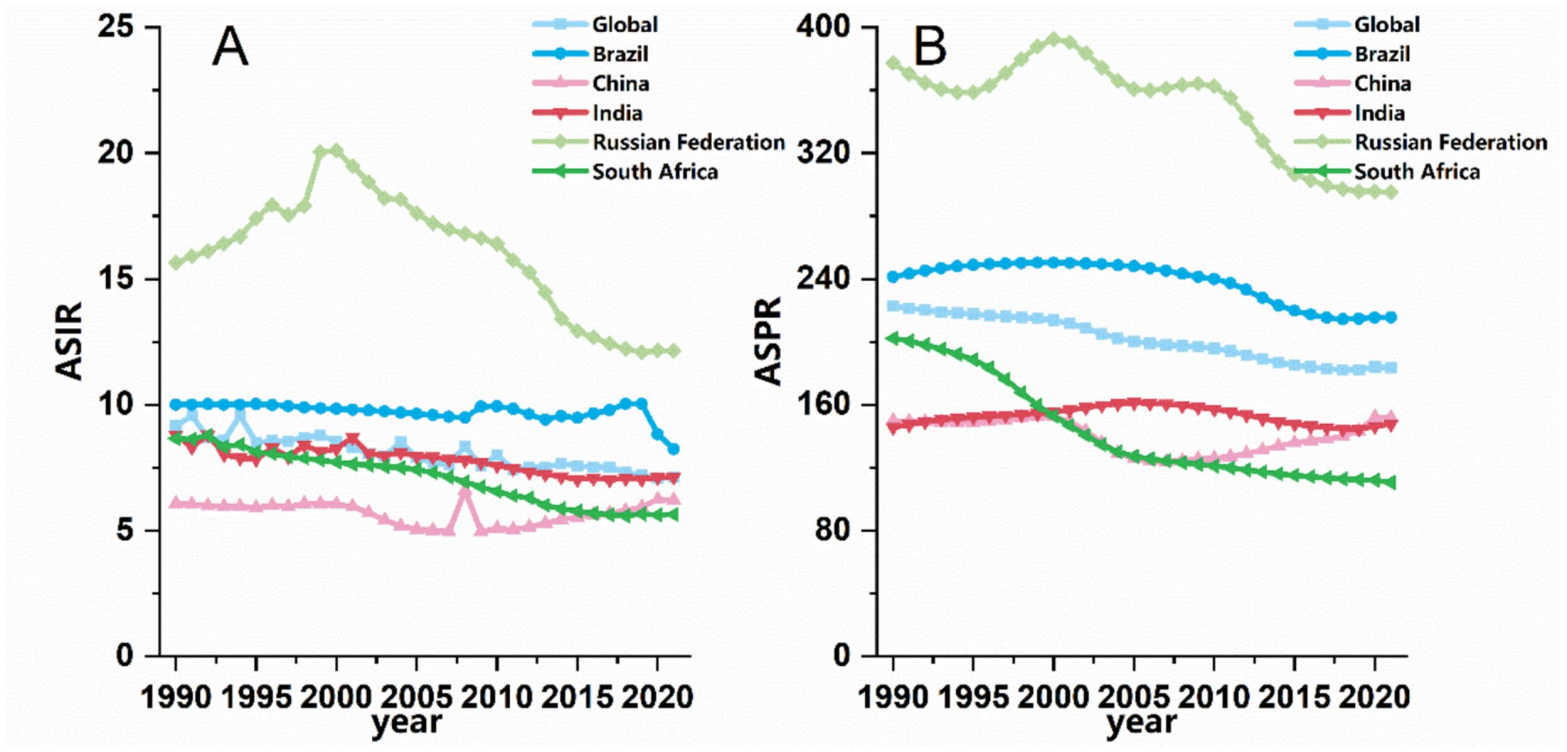
Trends in **(A)** ASIR and **(B)** ASPR of SCI in BRICS countries from 1990 to 2021.

Table 1 presents trends in the SCI burden across BRICS countries from 1990 to 2021. With the exception of Russia, the number of incident/prevalent cases increased in all BRICS countries, consistent with the global trend. On the contrary, the ASRs declined across all BRICS countries. Specific variations are detailed below (Table 1 and Figure 1).

**Table 1:** Number of cases and ASRs (per 100,000) for SCI across BRICS countries, 1990 and 2021.

Location	Measure	1990		2021		1990–2021 EAPC
		All-ages cases	ASR (per 100,000)	All-ages cases	ASR (per 100,000)	
		*n* (95% CI)	*n* (95% CI)	*n* (95% CI)	*n* (95% CI)	*n* (95% CI)
Global	Incidence	473666.32 (377726.29–598763.98)	9.16 (7.28–11.68)	574502.32 (440218.93–757444.99)	7.12 (5.48–9.36)	−0.81 (−0.93 – –0.69)
	Prevalence	10820146.34 (9937397.58–11841568.34)	222.7 (205.58–241.62)	15400682.49 (14009113.95–17075106.18)	183.56 (166.96–203.7)	−0.73 (−0.77 – –0.69)
Brazil	Incidence	14555.51 (11202.17–19035.69)	10.01 (7.7–13.16)	18838.26 (14504.61–24842.28)	8.24 (6.37–10.82)	−0.25 (−0.37 – –0.12)
	Prevalence	315116.41 (283580.53–348388.43)	241.33 (218.21–264.93)	528823.27 (482202.58–579840.91)	215.63 (195.84–237.47)	−0.52 (−0.64 – –0.39)
China	Incidence	69352.03 (54773.56–88357.18)	6.07 (4.76–7.84)	99363.45 (72456.32–136732.89)	6.21 (4.65–8.4)	−0.23 (−0.52 – 0.07)
	Prevalence	1694264.02 (1574087.24–1843869.31)	149.84 (139.72–162.12)	2766276.89 (2557986.11–3007580.51)	151.69 (140.39–164.98)	−0.34 (−0.6 – –0.07)
India	Incidence	58581.41 (44936.73–78111.64)	7.95 (5.91–10.87)	89419.14 (65898.77–124876.72)	6.73 (4.84–9.57)	−0.61 (−0.72 – –0.5)
	Prevalence	933244.17 (849298.51–1030416.95)	131.41 (119.72–144.41)	1841574.7 (1674695.41–2019845.95)	132.08 (120.23–145.42)	−0.13 (−0.22 – –0.03)
Russian Federation	Incidence	24125.19 (19002.75–30919.04)	15.65 (12.4–20.02)	18470.6 (14171.36–24561.85)	12.15 (9.44–15.97)	−1.23 (−1.63--0.82)
	Prevalence	627876.78 (578121.07–681667.58)	377.39 (347.47–409.43)	531501.51 (485297.12–581774.63)	295.16 (269.54–323.88)	−0.81 (−1.02 – –0.61)
South Africa	Incidence	3164.16 (2402.56–4286.49)	8.65 (6.59–11.62)	3380.03 (2586.21–4428.75)	5.64 (4.34–7.36)	−1.59 (−1.7 – –1.49)
	Prevalence	62647.91 (56900.65–71658.1)	202.31 (184.93–225.31)	65687.86 (60486.57–72141.42)	110.51 (101.97–121.28)	−2.15 (−2.39 – –1.91)

Incidence: China and India reported the highest absolute numbers of incident cases. In China, cases increased by 43.22%, from 69,352 to 99,363; in India, they increased by 52.56%, from 58,581 to 89,419 (This may be related to the large population base and severe aging of both countries, refer to the discussion section for more details.). In contrast, Russia witnessed a decline from 24,125 to 18,471 cases. Despite the rise in crude case numbers, the ASIR decreased in all BRICS countries from 1990 to 2021 (EAPC < 0). Both Brazil and Russia reported ASIRs higher than the global average (9.16 per 100,000 in 1990 and 7.12 in 2021). Brazil’s ASIR declined from 10.01 to 8.24 per 100,000, while Russia’s fell from 15.65 to 12.15 per 100,000. Notably, Russia and South Africa exhibited the most pronounced declines in ASIR, with EAPCs of −1.23 (95% CI: −1.63 to −0.82) and −1.59 (95% CI: −1.74 to −1.49), respectively (refer to the discussion section for possible explanations).

Prevalence: India and China showed the most substantial growth in prevalent cases, with rises of 97.33 and 63.27% from 1990 to 2021, respectively. By 2021, India had reached 1,841,575 prevalent cases, while China reported 2,766,277. Collectively, these two countries accounted for 29.92% of the global prevalent cases (15,400,682), and the total number across all BRICS countries represented 37.23% of the global burden. Despite these increases in absolute numbers, the ASPR declined across all BRICS countries over the study period. India experienced the smallest reduction (EAPC = −0.13), whereas South Africa showed the most marked decrease (EAPC = −2.15; 95% CI: −2.39 to −1.91). Potential interpretations of these trends are discussed in the subsequent section (Table 1 and Figure 1).

### Incidence and prevalence of SCI in BRICS countries by age- and sex-specific distributions

**Figure 2:**
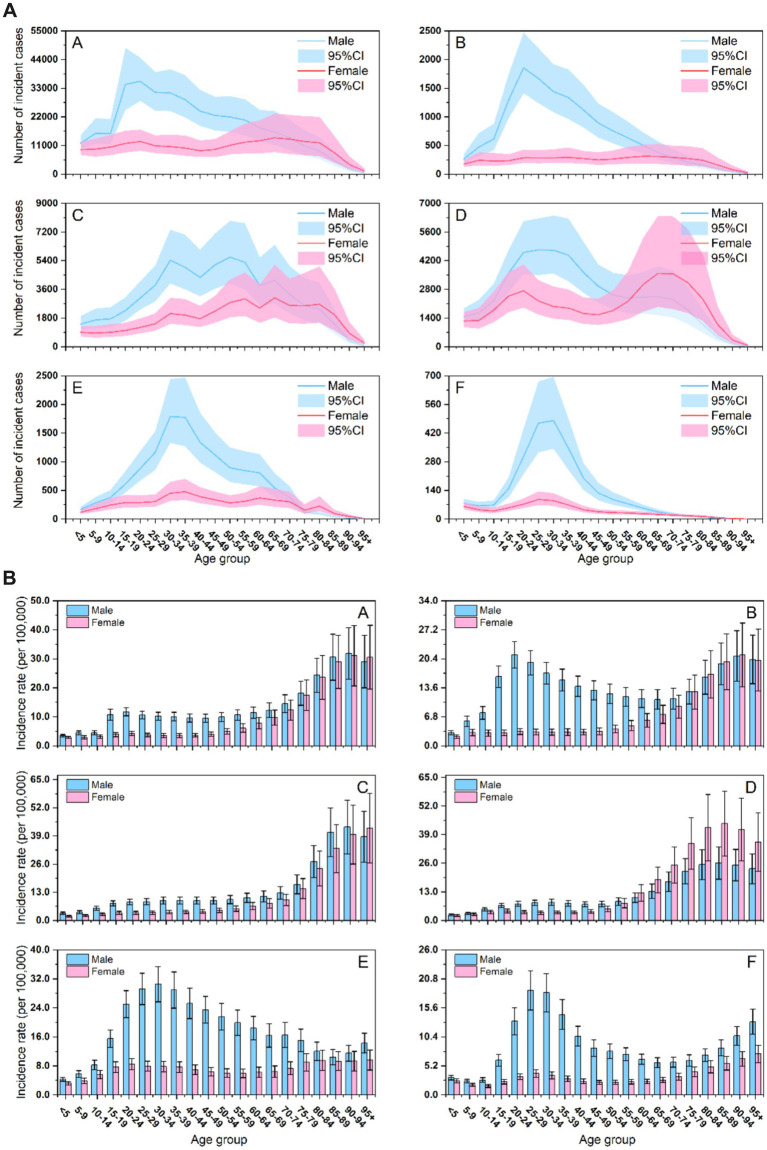
**(A)** Number of incident cases of SCI across age groups, sexes, and BRICS countries. (A) Worldwide; (B) Brazil; (C) China; (D) India; (E) Russian Federation; (F) South Africa. **(B)** Age-specific incidence rates of SCI across age groups, sexes, and BRICS countries. (A) Worldwide; (B) Brazil; (C) China; (D) India; (E) Russian Federation; (F) South Africa.

Figure 2 reveals the age- and sex-specific distributions in 2021 SCI across BRICS countries, presenting both the absolute number of incident cases (Figure 2A) and age-specific incidence rates (Figure 2B).

The number of incident cases (Figure 2A) showed unimodal or bimodal age distributions in all countries, with consistently higher peaks among males, reflecting a greater absolute burden of SCI in male. Cross-national variations emerged: in Brazil, Russia, and South Africa, cases among males surged to an early peak in young adulthood (ages 20–35) before declining rapidly. Chinese males displayed a distinct bimodal distribution, characterized by an initial peak around age 30 and a second, more pronounced peak around age 55. Although female case counts were generally lower and increased more gradually with age, females in China and India maintained elevated levels over a broader age range compared to other BRICS countries.

Age-specific incidence rates (Figure 2B) revealed two divergent epidemiological patterns. Brazil, Russia, and South Africa were characterized by an early peak with late rebound, with rates climbing rapidly to a maximum in young adulthood (ages 20–35), declined sharply, and rising again modestly after age 75. In contrast, China and India showed a late-peak pattern, with incidence rates increasing progressively with age and reaching a maximum in older adulthood. Sex disparities were pronounced: the peak incidence rate was substantially higher among males across most age groups and countries, reinforcing their high-risk status. Exceptionally high rates among young males in Brazil, Russia, and South Africa represent a critical public health concern. India was a notable exception, with peak incidence higher in females and sustained over a wider age range. In China, a steep rise in incidence among middle-aged and older females identified another vulnerable subgroup.

**Figure 3:**
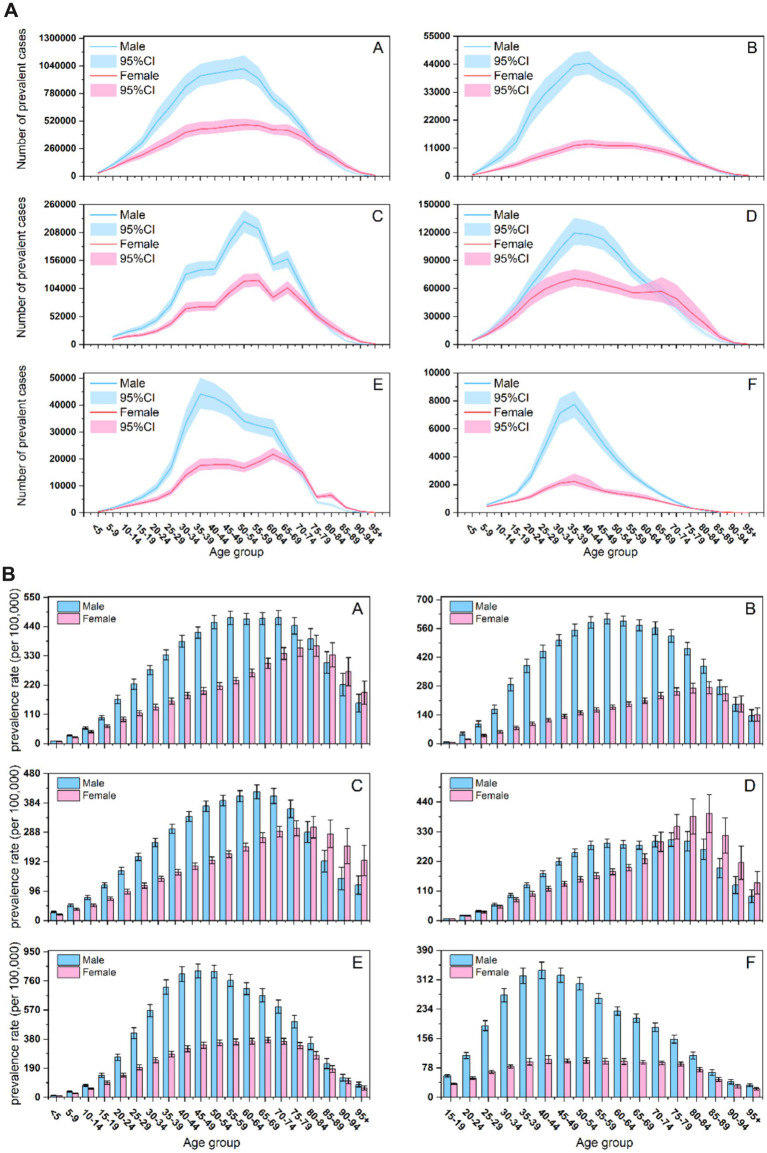
**(A)** The number of prevalent cases of SCI across age groups, sexes, and BRICS countries. (A) Worldwide; (B) Brazil; (C) China; (D) India; (E) Russian Federation; (F) South Africa. **(B)** The age-specific prevalence rate of SCI across age groups, sexes, and BRICS countries. (A) Worldwide; (B) Brazil; (C) China; (D) India; (E) Russian Federation; (F) South Africa.

Figure 3 shows the number of prevalent cases (Figure 3A) and age-specific prevalence rates (Figure 3B) of SCI in BRICS countries in 2021.

Prevalent case numbers (Figure 3A) followed an inverted U-shaped age distribution in all countries. While males predominated at the distribution peak, notable sex disparities emerged in older adults: case numbers were similar between sexes in Brazil, higher among older adult(s) females in China and India, and consistently higher among older adult(s) males in Russia and South Africa. China displayed a unique bimodal distribution, with peaks in 50–54 and 65–69 age groups.

The age-specific prevalence rates (Figure 3B) also exhibited an inverted U-shaped, with peak rates occurring later in females than in males across all countries. Geographic variations were apparent in older adults: prevalence rates were similar between sexes in Brazil, significantly higher among older adult(s) females in China and India, and higher among older adult(s) males in Russia and South Africa. The age of peak prevalence varied nationally, occurring earliest in South Africa (approximately 40–44 years), followed by Russia (45–50 years), and latest in China (60–64 years).

### Joinpoint regression analysis of ASIR and ASPR for SCI in BRICS countries, 1990–2021

From 1990 to 2021, significant disparities were observed in the decline of ASIR across genders and BRICS countries (all AAPC < 0 with statistical significance, except China), aligning with the global trend. South Africa experienced the most pronounced decline (AAPC = −1.59; 95% CI: −1.68 to −1.36), while China showed only a modest, non-significant decrease (AAPC = −0.23; 95% CI: −0.52 to 0.07). Notably, the decline in ASIR was more substantial among females than males in Brazil, China, and South Africa (Table 2).

**Table 2:** Joinpoint regression analysis of trends in the burden of disease for SCI in the BRICS countries, 1990–2021.

Location	Gender	ASIR		ASPR	
		AAPC	95%CI	AAPC	95%CI
Global	Both	−0.8802*	(−1.1497 – –0.6311)	−0.6059*	(−0.6213 – –0.5926)
	Male	−0.8139*	(−0.9395 – –0.6867)	−0.6463*	(−0.6601 – –0.6325)
	Female	−0.7894*	(−1.032 – –0.5486)	−0.5334*	(−0.5471 – –0.5215)
Brazil	Both	−0.2486*	(−0.4153 – –0.0827)	−0.3627*	(−0.3719 – –0.3535)
	Male	−0.1598	(−0.3683–0.0491)	−0.2271*	(−0.2364 – –0.2178)
	Female	−0.6243*	(−0.7706 – –0.4734)	−0.6991*	(−0.7102 – –0.6886)
China	Both	−0.2255	(−0.5193 – 0.0737)	0.0903*	(0.0196 – 0.1506)
	Male	−0.0923	(−0.3415 – 0.1634)	0.1603*	(0.1118 – 0.2144)
	Female	−0.4240*	(−0.8328 – –0.0001)	−0.0686*	(−0.1335 – –0.0122)
India	Both	−0.6115*	(−0.7282 – –0.4922)	0.0184*	(0.0049 – 0.03)
	Male	−0.6647*	(−0.7958 – –0.5298)	0.0457*	(0.0307 – 0.0591)
	Female	−0.5724*	(−0.7116 – –0.4319)	−0.0051	(−0.0158 – 0.0039)
Russian Federation	Both	−1.2287*	(−1.7896 – –0.6621)	−0.7853*	(−0.8244 – –0.7465)
	Male	−1.3353*	(−1.9161 – –0.749)	−0.9059*	(−0.945 – –0.853)
	Female	−0.8384*	(−1.3988 – –0.2584)	−0.5399*	(−0.5701 – –0.5103)
South Africa	Both	−1.5929*	(−1.7426 – –1.4395)	−1.9250*	(−1.9421 – –1.9048)
	Male	−1.5237*	(−1.6845 – –1.3599)	−1.7627*	(−1.7784 – –1.7427)
	Female	−2.0102*	(−2.1703 – –1.8465)	−2.3850*	(−2.4154 – –2.3521)

* Indicates *p* < 0.05.

In contrast, trends in ASPR diverged across BRICS countries. China (AAPC = 0.09, *p* < 0.05) and India (AAPC = 0.02, *p* < 0.05) showed a slight increase in ASPR, driven primarily by rising rates among males. Brazil also experienced a decline (AAPC = −0.25, *p* < 0.05), though it was less marked than the global average (AAPC = −0.61; *p* < 0.05). Conversely, Russia (AAPC = −0.79, *p* < 0.05) and South Africa (AAPC = −1.93, *p* < 0.05) demonstrated steeper declines than both the global average and other BRICS countries. The underlying drivers of these differential trends merit further exploration and are discussed in a subsequent section.

### Etiology of SCI across BRICS countries

**Figure 4:**
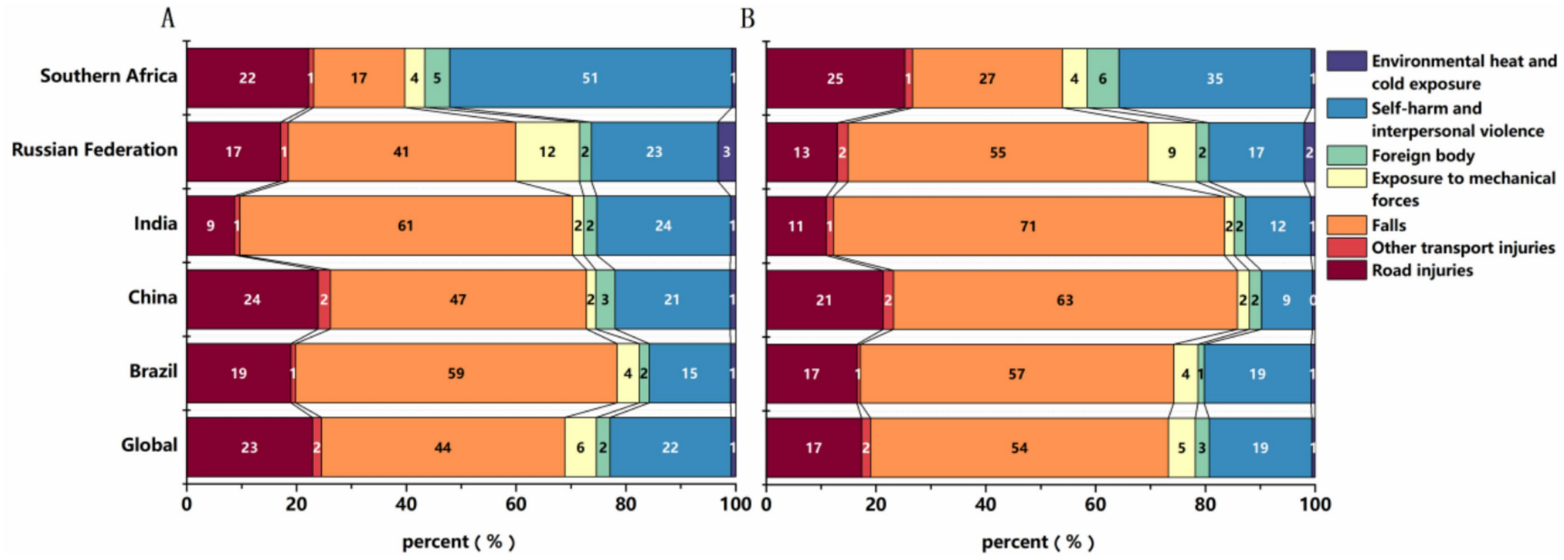
Etiology of SCI: proportional contributions of major causes to the ASIR in 1990 **(A)** and 2021 **(B)**.

The proportional contributions of different causes to ASIR of SCI globally and in BRICS countries in 1990 and 2021 were presented in Figure 4. The results indicated that falls, road injuries, self-harm and interpersonal violence were among the leading causes of SCI.

Falls were the predominant cause across most BRICS countries. In 2021, they comprised over 50% of the global ASIR of SCI. Among the BRICS countries, India reported the highest proportion, exceeding 70%, while South Africa demonstrated a notably lower share of SCI due to falls (27%). Instead, self-harm and interpersonal violence collectively were responsible for 35% of SCI cases in South Africa (see the Discussion section for possible reasons.).

The relative contributions of different causes shifted substantially between 1990 and 2021. The proportion of SCI caused by falls increased by more than 10% in all BRICS countries except Brazil. Conversely, the proportion resulting from self-harm and interpersonal violence generally declined, while other causes remained relatively stable (see Figure 4). The underlying drivers of these trends, including potential policy influences, are examined in the Discussion.

### Forecast of SCI burden in BRICS countries, 2022–2031

The optimal ARIMA models for predicting ASIR of SCI, selected based on the lowest AIC and BIC values. Country-specific models included ARIMA (0,1,2) for Brazil, ARIMA (1,0,0) for China, ARIMA (0,1,1) for India, ARIMA (0,2,2) for Russia, and ARIMA (2,1,0) for South Africa. Residual diagnostics using the Ljung–Box test showed no significant autocorrelation at lag 6 or lag 12 (all *p* > 0.05), supporting model adequacy and confirming that residuals approximate white noise (Table 3). All models demonstrated high predictive accuracy, with low MAPE, MAE, and RMSE.

**Table 3:** Optimal ARIMA models and forecast accuracy metrics for SCI burden in BRICS countries.

Indicators	Location	ARIMA(p,d,q)	AIC	BIC	RMSE	MAE	MAPE (%)	Chi-square values of lag 6	p	Chi-square values of lag 12	P
ASIR	Global	ARIMA(3,1,1)	19.24	27.85	0.258	0.218	2.669	0.331	0.999	6.022	0.915
	Brazil	ARIMA(0,1,2)	2.93	7.23	0.222	0.114	1.212	3.372	0.761	9.275	0.679
	China	ARIMA(1,0,0)	34.73	39.12	0.377	0.252	4.516	2.630	0.854	4.840	0.963
	India	ARIMA(0,1,1)	−1.77	2.53	0.209	0.163	2.223	6.883	0.332	15.537	0.213
	Russian Federation	ARIMA(0,2,2)	51.2	55.4	0.490	0.316	1.853	2.240	0.896	5.151	0.953
	Southern Africa	ARIMA(2,1,0)	−43.09	−37.36	0.104	0.071	1.004	2.047	0.915	5.642	0.933
ASPR	Global	ARIMA(2,1,0)	69.02	74.76	0.626	0.400	0.204	1.261	0.974	4.495	0.973
	Brazil	ARIMA(3,2,0)	33.13	38.73	0.349	0.206	0.090	2.431	0.876	8.067	0.780
	China	ARIMA(0,2,0)	137.05	138.45	2.225	1.228	0.868	1.452	0.963	1.876	0.999
	India	ARIMA(0,2,0)	36.32	37.72	0.416	0.273	0.203	3.893	0.691	7.594	0.816
	Russian Federation	ARIMA(2,1,1)	112.82	119.99	1.150	0.900	0.262	1.208	0.977	7.254	0.840
	Southern Africa	ARIMA(3,2,0)	52.31	57.91	0.480	0.300	0.212	9.548	0.145	16.394	0.174

For ASPR, the optimal ARIMA models were ARIMA (3,2,0) for Brazil, ARIMA (0,2,0) for China, ARIMA (0,2,0) for India, ARIMA (1, 2) for Russia, and ARIMA (3,2,0) for South Africa. The predictive performance metrics for both ASIR and ASPR forecasts are summarized in Table 3.

**Figure 5:**
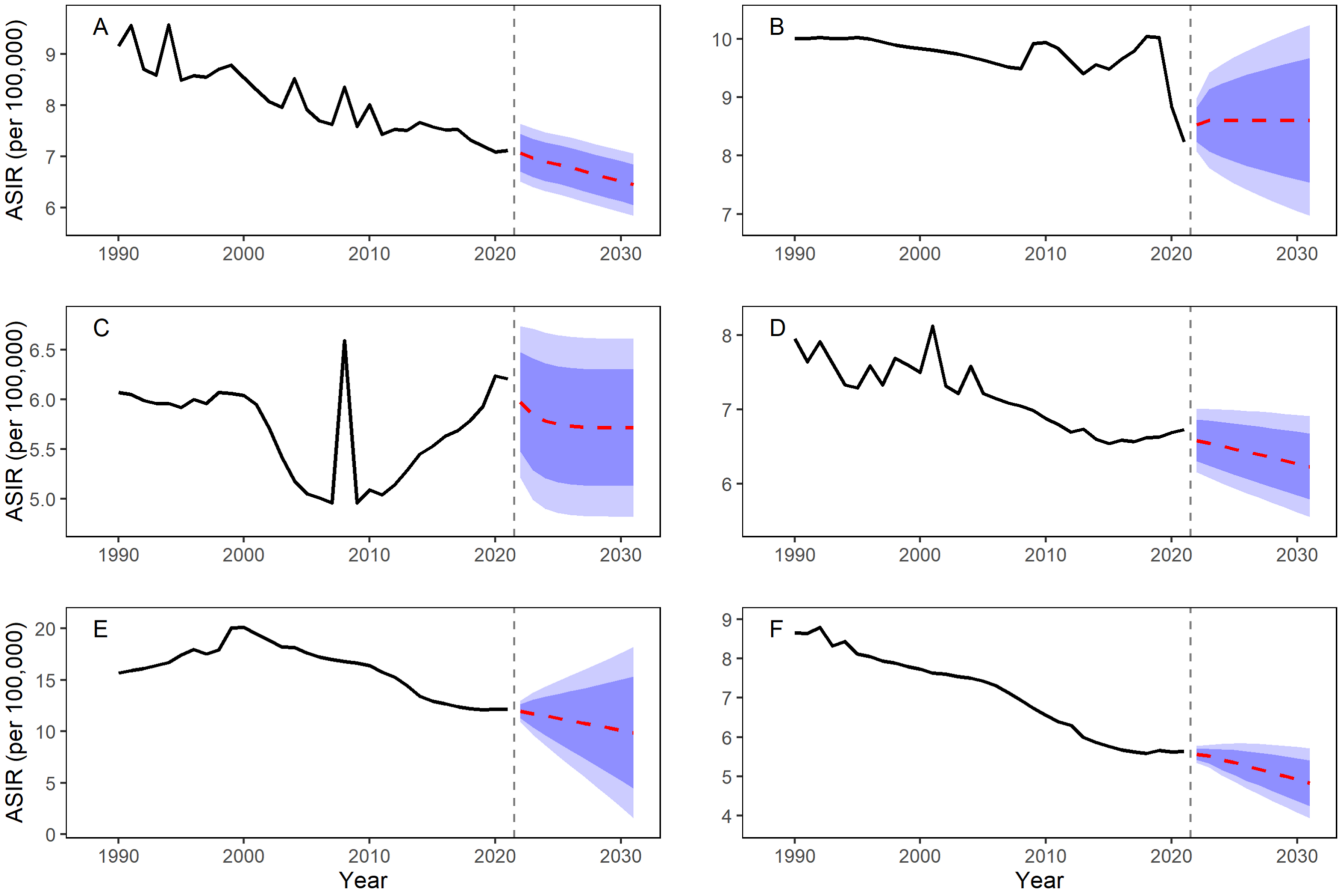
Forecasted ASIR for SCI in BRICS countries, 2022–2031. **(A)** Worldwide; **(B)** Brazil; **(C)** China; **(D)** India; **(E)** Russian Federation; **(F)** South Africa.

**Figure 6:**
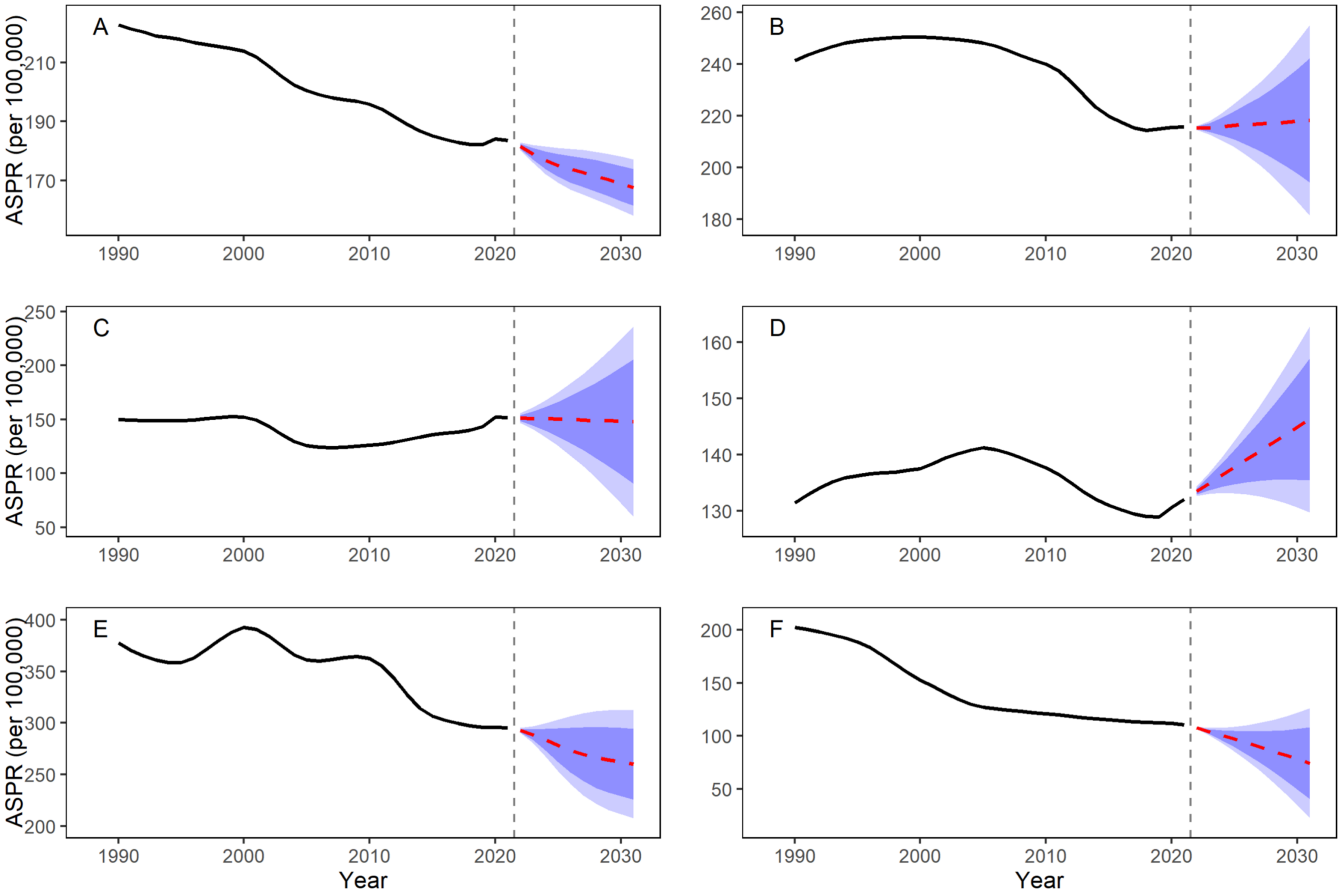
Forecasted ASPR for SCI in BRICS countries, 2022–2031. **(A)** Worldwide; **(B)** Brazil; **(C)** China; **(D)** India; **(E)** Russian Federation; **(F)** South Africa.

The ARIMA model demonstrated robust predictive performance across both training and test sets, as indicated by standard accuracy metrics. Projections based on the fitted disease burden from 1990 to 2021 indicate a general decline in ASIR and ASPR across most BRICS countries during 2022–2031; however, heterogeneous patterns trends observed for ASR, with increases projected in some countries (Table 4 and Figures 5, 6).

**Table 4:** Forecasted ASIR and ASPR for SCI in BRICS countries, 2022–2031.

Indicators	Year	Predicted value(per 100,000)					
		Global	Brazil	China	India	Russian Federation	Southern Africa
ASIR	2022	7.07	8.53	5.97	6.58	11.96	5.56
	2023	6.97	8.61	5.85	6.55	11.73	5.52
	2024	6.89	8.61	5.79	6.51	11.50	5.42
	2025	6.84	8.61	5.75	6.47	11.27	5.35
	2026	6.78	8.61	5.73	6.43	11.04	5.26
	2027	6.71	8.61	5.73	6.39	10.81	5.18
	2028	6.64	8.61	5.72	6.35	10.58	5.09
	2029	6.58	8.61	5.72	6.31	10.35	5.01
	2030	6.51	8.61	5.72	6.27	10.12	4.92
	2031	6.45	8.61	5.72	6.23	9.89	4.83
Rate of change (%)		−8.83	0.87	−4.33	−5.31	−17.36	−13.12
ASPR	2022	181.55	215.35	151.32	133.50	292.74	107.64
	2023	179.07	215.32	150.95	134.92	288.70	104.09
	2024	176.86	215.72	150.58	136.34	283.67	100.55
	2025	175.16	216.25	150.21	137.76	278.43	97.12
	2026	173.86	216.60	149.84	139.18	273.60	93.59
	2027	172.74	216.79	149.47	140.60	269.59	89.85
	2028	171.60	217.03	149.10	142.02	266.46	85.96
	2029	170.37	217.40	148.73	143.44	264.05	82.06
	2030	169.04	217.82	148.36	144.86	262.03	78.19
	2031	167.65	218.20	147.99	146.28	260.05	74.32
Rate of change (%)		−7.66	1.32	−2.20	9.57	−11.17	−30.96

Notably, Brazil’s ASIR is projected to show minimal change throughout the forecast period (predicted value approximately 8.61 per 100,000 in 2031). In contrast, Russia is projected to experience the largest decline (−17.36%). Despite this decrease, Russia’s ASIR in 2031 is projected to remain the highest among BRICS at 9.89 (95% CI: 1.58–18.19). South Africa is projected to maintain the lowest ASIR (4.83 per 100,000).

Divergence is more pronounced in ASPR trends: Brazil’s ASPR is projected to increase to 218.20 per 100,000 by 2031, and India’s to 146.28 per 100,000. Meanwhile, South Africa’s ASPR is expected to decline by 30.96%, falling to 74.32 per 100,000 in 2031.

## Discussion

The evolutionary trend of SCI disease burden across BRICS countries is influenced by a complex interplay of socioeconomic, demographic, and health policy factors, which reflect both challenges and opportunities in rapid development. To elucidate these patterns, this study utilized GBD 2021 data to systematically compare and analyze the epidemiological characteristics and leading causes of SCI in BRICS countries from 1990 to 2021, and projected the SCI burden through 2031. Below we discuss trends across demographic subgroups, etiological profiles, and study limitations.

### Long-term trends and drivers of SCI burden

ASRs of SCI consistently declined across BRICS countries from 1990 to 2021 (EAPC<0), suggesting improved injury prevention and clinical management. However, the absolute number of incident and prevalent cases continued to rise, primarily driven by population growth and aging. For instance, China and India experienced substantial increases in the numbers of incident cases (43.22 and 52.56%, respectively), alongside decreases in ASIR (EAPC = -0.23 in China and EAPC = -0.61 in India). This pattern indicates that the beneficial effects of public health interventions (e.g., China’s criminalization of drunk driving in 2011, and India’s enhanced building safety regulations) may have partially offset the demographic pressures of a growing and aging population (e.g., China’s population aged ≥65 years increased from 5.6% in 1990 to 13.5% in 2021). Similar trends between crude and ASRs have been observed in other transitioning economies (5, 6), indicating that health systems can achieve progress in injury control despite demographic headwinds.

South Africa achieved significant reductions in ASIR (EAPC = −1.59) and ASPR (EAPC = −2.59). This encouraging trend coincided with the implementation of its National Injury Prevention Strategy (2016–2021), which targeted major SCI causes such as violence and road injuries, suggesting a potential positive effect of coordinated policy action (12). Among BRICS countries, Russia uniquely exhibited a decline in the absolute number of cases. This decline may be linked to structural improvements in its trauma care system, such as expanded tertiary trauma center coverage (78% by 2012), coupled with a marked reduction in alcohol consumption (e.g., per capita alcohol intake decreased from 15.7 liters in 2003 to 11.1 liters in 2018) (12, 16). These factors may have collectively reduced alcohol-related violence and traffic incidents, major contributors to SCI (12). This study also found that the ASRs in Russia and Brazil consistently exceeded the global average throughout the study period, underscoring the need for targeted interventions addressing local etiologies of SCI such as occupational exposures (e.g., a 22% injury rate in the construction sector) and interpersonal violence (e.g., a homicide rate of 23.6 per 100,000 in 2021).

It is worth noting that data quality variations further complicate cross-national comparisons; well-established SCI registries in high-income countries likely capture more cases than underreported data in some BRICS countries (23). To address these gaps, we recommend establishing a unified BRICS SCI registry consortium to standardize diagnostic criteria, improve the quality of data collection, and facilitate stratified interventions for high-risk populations, thereby improving the quality and accessibility of care and rehabilitation services. Complementing this, a regional trauma quality improvement network could provide an innovative paradigm for global health governance. Such infrastructure has proven instrumental in high-income countries for guiding targeted public health actions and could significantly enhance the accuracy of burden estimates and efficacy of interventions across BRICS. For instance, the incidence rates of SCI have decreased in Europe and high-income Asia-Pacific regions primarily due to preventive legislation, while comprehensive rehabilitation systems have helped to mitigate the long-term burden of prevalent cases (24).

### Analysis of demographic differences in the burden of SCI in the BRICS countries

Gender disparities and risk factors: Males in BRICS countries face a significantly higher risk of SCI than females, particularly within the 20–40 age group, a finding consistent with prior research (6). This disparity is largely driven by gender-based labor divisions and differential exposure to risks: males disproportionately engage in high-risk occupations (e.g., mining, construction) and exhibit higher rates of alcohol abuse (25). In Brazil and South Africa, inadequate occupational protections contribute to an heightened burden of SCI among young males (11), while in Russia, high rates of alcohol abuse among males increase susceptibility to violence and traffic accidents (16). These trends reflect broader patterns observed in many low- and middle-income countries (LMIC), where industrialization and socioeconomic transitions exacerbate occupational and behavioral risks among males. Therefore, occupational health education should be enhanced for high-risk male groups, with preventive measures aimed at avoiding high-risk behaviors and hazardous environments to reduce the likelihood of traumatic SCI development.

Age profiles and etiological shifts: The age distribution of SCI cases also reveals distinct epidemiological patterns, shaped by differing primary injury mechanisms. Brazil, Russia, and South Africa show peaks in young adulthood (20–35 years), with causative factors including street violence, motorcycle crashes, and interpersonal conflicts (12). In contrast, China and India experienced a later peak (50–54 age group), likely reflecting a higher burden of aging-related etiologies such as falls, degenerative spinal disorders, and chronic diseases like diabetic neuropathy (13). Among older adults, especially females, face elevated risks of falls, fractures, and SCI due to age-related bone density loss (26, 27). This risk is further compounded by a high prevalence of degenerative spinal disorders, such as spinal stenosis and herniated discs (28). Therefore, enhancing health management for older females with measures such as bone density screening, fall prevention, and expanded home-based care is crucial to reducing secondary injuries in aging communities.

### The causes of SCI variation and their socioeconomic associations

Falls, road injuries, self-harm and interpersonal violence were the leading causes of SCI across BRICS countries, though their relative contributions varied geographically. Falls accounted for more than 70% of SCI cases in India, a markedly high proportion likely driven by agricultural risks, underdeveloped rural infrastructure, and inadequate geriatric care (12). This figure surpasses that of many other LMICs and underscores how infrastructural gaps profoundly influence the SCI burden. In China, falls also represent a major concern, influenced by factors such as low helmet-wearing rates (<30%) among rural e-bike riders and design shortcomings in urban public facilities (5, 12). The significant burden of road injuries in these countries aligns with broader global trends. Previous studies suggest traffic-related injuries are more frequent in countries with higher Socio-demographic Index (SDI) compared to those with lower SDI (29), indicating that enhanced road safety measures must keep pace with economic development.

South Africa presents a distinct profile. A decline in SCI due to self-harm and interpersonal violence (from 51% in 1990 to 35% in 2021), may reflect the positive impact of recent social stabilization initiatives. Nevertheless, persistent social inequality and high crime rates point to an ongoing need for targeted interventions (12). Despite improvements, South Africa’s burden remains high relative to global averages, underscoring the lingering effects of socioeconomic disparity, a challenge also seen in other high-inequality settings.

The proportion of SCI caused by falls increased by more than 10% in all BRICS countries except Brazil between 1990 and 2021, suggesting that safety regulations have lagged behind rapid population aging and urban expansion (5, 13). This trend mirrors the historical experience of high-income countries and calls for multisectoral collaboration, improved urban planning, and stronger healthcare policies for older adults (6, 11).

Etiological patterns also diverged by age. Traumatic SCI, commonly resulting from motor vehicle accidents (43.2%) and falls (34.2%), was more frequent among younger and older populations. In contrast, nontraumatic SCI (e.g., degenerative and metabolic causes) became increasingly prevalent with advancing age (30). This clear distinction highlights the need for etiology-specific prevention strategies tailored to specific age groups.

### Projected overall downward trend in SCI disease burden in BRICS countries from 2022 to 2031

Using ARIMA models, a well-established method for time series forecasting, we projected a general decline in ASRs across BRICS countries from 2022 to 2031, though country-level trajectories will vary. Brazil, China, and India are projected to show slower declines or even slight increases in ASR, indicating that current prevention strategies may be inadequate to fully offset demographic shifts and emerging risk factors. These trends underline the need for strengthened interventions, such as promoting electric bicycle helmet use and enhancing building safety standards (5, 9). In contrast, South Africa is projected to achieve substantial reductions by 2031, with an estimated 13.12% decrease in ASIR and 30.96% decrease in ASPR. This success could serve as an instructive model for other LMICs seeking to reduce injury-related burden through integrated policy action.

A key limitation of these projections is that the models do not account for exogenous shocks (e.g., pandemics, social unrest, or sudden policy shifts). This underscores the value of developing adaptive prediction frameworks that can better account for stochastic public health events.

### Study limitations and outlook

This study has several limitations that warrant consideration. First, as the GBD estimates are modeling-based, they may not fully reflect the actual disease burden, especially in regions where primary data are scarce. Second, due to limitations in data availability and granularity, we were unable to examine subnational variations (e.g., urban–rural disparities in China), or to perform a more detailed categorical analysis of causes (e.g., occupational vs. domestic falls). Finally, the ARIMA models did not incorporate external variables such as health emergencies or abrupt policy changes, which could influence forecast accuracy.

Future research should aim to fill these gaps by strengthening data collection accuracy through targeted epidemiological surveys, incorporating multi-source data and machine learning methods to improve model reliability, and enabling more granular analyses of the SCI burden across key subgroups and etiology-specific contexts.

## Conclusion

The evolution of the SCI burden across BRICS countries is shaped by overlapping demographic transition, policy efficacy, and structural societal challenges. Our analysis indicates that although the ASR of SCI generally declined from 1990 to 2021, the absolute number of cases continues to rise, driven by population growth and aging. Furthermore, males and older individuals consistently faced higher SCI risks than females and younger adults.

Integrating targeted prevention, optimized healthcare resources, and evidence-based policy reforms could further reduce the SCI burden across BRICS countries. To achieve this, future research perhaps can focus on improving data quality through standardized collection methods, establishing a unified surveillance system to monitor SCI trends, formulating tailored preventive measures for specific injury causes and demographic groups, and strengthening international cooperation to promote equitable access to preventive and rehabilitative care. Collectively, these strategies outline a concrete approach to mitigating the global impact of SCI.

## Data Availability

The datasets used in this study were derived from the GBD 2021 database and are accessible via the Global Health Data Exchange (GHDx).
